# Examining Comorbid Psychopathology Symptoms as Predictors of Family Based Treatment for Adolescents With Anorexia Nervosa and Atypical Anorexia Nervosa in a Real‐World Setting

**DOI:** 10.1002/erv.70124

**Published:** 2026-05-02

**Authors:** Daniel Wilson, Hilary Grimmer, Renata A. Mendes, Jenna Irwin, Julie Atherton, Richard Litster, Jacinda White, Natalie J. Loxton

**Affiliations:** ^1^ Child and Youth Mental Health Service Eating Disorder Program Children's Hospital Queensland Brisbane Queensland Australia; ^2^ Child Health Research Centre University of Queensland Brisbane Queensland Australia; ^3^ School of Applied Psychology Griffith University Nathan Queensland Australia

**Keywords:** adolescents, anorexia nervosa, eating disorders, family based treatment, predictors

## Abstract

**Background:**

For adolescents with Anorexia Nervosa (AN) or Atypical Anorexia Nervosa (AAN), Family Based Treatment (FBT) is an effective treatment. However, outcomes remain suboptimal, making investigations into predictors of outcome important. Most prior research into FBT has focussed on parental and family factors as predictors.

**Objective:**

The current study aimed to identify predictors of FBT for adolescents with AN and AAN in a real‐world setting, specifically focussed on comorbid patient psychopathology as a predictor of outcome.

**Method:**

A prospective cohort of 135 young people engaging in manualised FBT (female = 92.6%, age = 14.33 years, SD = 1.54, range 11–17) at a public outpatient child and youth eating disorder (ED) service were evaluated. Measures of ED and comorbid psychopathology and BMI outcomes were evaluated pre‐ and post‐treatment.

**Results:**

Results showed that age was a predictor of drop‐out and weight gain, with older age associated with higher chance of drop‐out and less weight gain. No comorbid psychopathology measure predicted drop‐out or treatment outcomes.

**Discussion:**

Findings highlight the potential role of age in treatment non‐completion and weight regain in FBT, suggesting the potential need for developmental considerations to FBT among this group.

## Highlights

- Age predicted drop‐out among adolescents with anorexia nervosa and atypical anorexia nervosa engaged with family‐based treatment.
- Baseline BMI and age predicted end of treatment BMI outcomes.
- All measures of ED and comorbid psychopathology as well as BMI centile improved post treatment.

## 1 Introduction

Eating disorders (EDs) can be severe and enduring psychiatric disorders, associated with high mortality, significant medical and psychological comorbidity, and considerable global disease burden for the individuals and their carers (van Hoeken and Hoek 2020; Wilson, Krishnamorthy, et al. 2025). Early and effective intervention is important for EDs, as the prevalence rates have increased over 2 decades (Galmiche et al. 2019), alongside earlier age of onset (Herpertz‐Dahlmann and Dahmen 2019). Family Based Therapy (FBT) is the frontline recommended treatment by the National Institute for Health and Care Excellence (NICE) guidelines (NICE: 2020) for adolescent Anorexia Nervosa (AN). FBT is the most well studied treatment for adolescents, and has demonstrated effectiveness for AN, as well as for adolescents with Atypical Anorexia Nervosa (AAN). FBT prioritises early behavioural change, focussing primarily empowering parents to achieve weight restoration and disrupting eating disorder behaviours in the first phase of treatment, placing less focus on broader psychological difficulties. However, despite its empirical support, full remission rates are less than 50%, highlighting the need to better understand factors that influence treatment response and explore whether treatment may be enhanced by adapting existing procedures to address these barriers (J. Lock and Le Grange 2019).

A recent scoping review into FBT for AN identified several patient‐level factors associated with positive outcomes, including younger age and shorter duration of illness; fewer hospitalisations or previous treatments; early weight gain; and lower food‐related obsessionally (Gorrell et al. 2022). Whilst informative, one of the limitations of existing identified predictors is that they are either unmodifiable at initial assessment (i.e., age, duration of illness, prior treatment), reflect an existing target of FBT (i.e., early weight gain), or are highly correlated with exiting ED symptoms (i.e., food related obsessionally is highly correlated with dietary restraint; Wilson et al. 2025). This suggests that FBT may already be optimised to target the identified predictors (e.g., early weight gain). As such, these findings offer limited guidance for how treatment itself might be adapted to improve outcomes. Exploring factors beyond those identified in prior research offers a promising avenue for improving FBT outcomes.

Comorbid psychiatric symptomology is one such potential factor. Comorbidity is highly prevalent among young people with EDs, and there is emerging evidence that it may impact treatment length (Lim et al. 2023; Wilson, Krishnamorthy, et al. 2025). However, comorbidity has been relatively underexplored as a predictor of FBT compared to other patient‐ and parent‐level variables. Existing findings are also inconsistent. For example, in a recent large study in an adolescent AN sample, Bentz et al. (2025) found that a diagnosis of a comorbid emotional or behavioural disorder predicted less weight gain throughout FBT and a greater chance of longer treatment duration. In contrast, Datta et al. (2023) used a binary predictor (no comorbid diagnosis vs. greater than one comorbid diagnosis) and found no effect on FBT outcomes across a large sample of adolescents with AN. Interpretation of these results is limited by the aggregation of diverse comorbid diagnoses into broad categories making it unclear how, and to what extent, comorbid psychopathology impacts FBT outcomes. Clarifying the role of comorbid symptoms and diagnoses in FBT outcomes is therefore essential, particularly given the availability and promise of established evidence‐based treatments for many comorbid conditions (e.g., anxiety disorders, depression, obsessive‐compulsive disorder).

In parallel, a larger body of established work has examined parent and family factors that influence FBT outcomes and potential benefit of addressing these with treatment adaptations. For example, higher levels of expressed emotion (specifically higher parental criticism) and poor parental self‐efficacy have been associated with poorer FBT outcomes (Gorrell et al. 2022; McCord et al. 2025). As such, several adjunct parent‐focused emotion coaching interventions has been trialled, appearing to improve FBT outcomes in these cases (Aarnio‐Peterson et al. 2024; J. D. Lock et al. 2024). Similarly, adjunctive treatment targeting non‐ED psychopathology in adolescents also shows promise (e.g., Hurst and Zimmer‐Gembeck 2019). However, targeting comorbid psychopathology of young people would represent a significant departure from the existing principles of FBT, which focusses on empowering parents to normalise eating behaviours and weight restoration in their child. There could be a risk that prioritising comorbid psychopathology may be iatrogenic through reducing focus on the main tasks of FBT. Therefore, an empirical basis identifying which factors present a barrier to FBT is required to inform any potential rationale for altering existing FBT protocols (Pedersen et al. 2024; Richards et al. 2023). In this study, we aim to explore whether comorbid psychiatric symptoms predict FBT outcome among young people with AN and AAN in an outpatient adolescent EDs clinic.

## 2 Method

### 2.1 Participants

**FIGURE 1:**
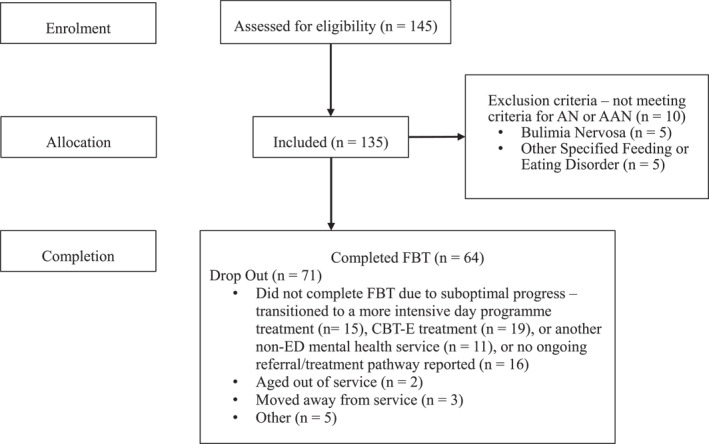
Participant flow during treatment.

This prospective cohort study involved 135 young people engaging in FBT, and (female = 92.6%, *M*_age_ = 14.33 years, SD = 1.54, range 11–17) at a public outpatient specialist child and youth ED service in Brisbane, Australia. The clinic caters to young people with a variety of EDs across the state. However, for this study's aims, only those with a diagnosis AN (36.3%) and AAN (63.7%) were included. A consort diagram of enrolment and attrition is shown in Figure 1. Diagnoses were made by an experienced psychiatrist with specialist training in child and youth EDs, with criteria strictly applied from the International Classification of Diseases and Related Health Problems (10th ed.; ICD‐10; World Health Organization 2016). Specifically, the 5^th^ centile BMI was used to differentiate AN from AAN, with AN also being diagnosed for individuals above the 5^th^ centile, if the persons weight was 15% below their premorbid centile if such data was available, consistent with ICD guidelines. As per ICD‐10, AAN was diagnosed if individuals demonstrated core features of AN, but their weight was above the 5^th^ centile and had no evidence of greater than 15% weight loss.

### 2.2 Procedure

Participants received FBT as per the treatment manual by J. Lock and Le Grange (2012). The intervention was delivered by accredited FBT clinicians including psychologists, social workers, nurse practitioners, and psychiatrists. All had training in FBT and general child and youth mental health and received regular monthly supervision. FBT consists of family sessions delivered over three phases, and is based on the premise that the child or adolescent with the ED is embedded in the family and therefore requires parental involvement in treatment in order to recover (J. Lock and Le Grange 2012). Phase I consists of weekly sessions focussed on empowering parents to take charge of nutrition and management of compensatory behaviours to facilitate weight restoration. Note, in AAN, weight restoration is often required to reverse starvation and medal instability however this is evaluated on a case‐by‐case basis. Once disordered eating behaviours are significantly reduced and the young person is weight restored to at least 90% expected body weight, the treatment moves to Phase II. This typically consists of fortnightly session, and involves a gradual return of the management of eating and movement back to the young person. Once expected body weight has been restored and eating behaviour normalised, the family transitions to Phase III of treatment. Phase III consists of sessions every 4–6 weeks and addresses more general issues of adolescent development (e.g., increasing autonomy, establishing boundaries), supporting the family to return age‐appropriate parenting and routines, and relapse prevention planning.

### 2.3 Measures

#### 2.3.1 Demographic and Clinical Variables

Young people answered items relating to their age and sex. Psychiatric diagnoses, weight and height were collected by the assessing clinician.

#### 2.3.2 Body Mass Index Centiles

BMI centiles were calculated using the young person's weight, height, and the Centre for Disease Control and Prevention growth charts (www.cdc.gov/growthcharts).

#### 2.3.3 Eating Disorder Examination Questionnaire

The global score from the EDE‐Q (Fairburn and Beglin 1994) was used to assess adolescent ED symptoms (dietary restraint, eating/weight/shape concerns). The adolescent version was chosen as it features developmental modifications with shortened time frames and simplified wording (Carter et al. 2001). The scale included 22 items, and it was scored on a 7‐point scale (0–6). It has demonstrated excellent internal reliability in previous studies (*α* = 0.97; Wilson, Krishnamorthy, et al. 2025).

#### 2.3.4 Patient Health Questionnaire‐9

The PHQ‐9 is a self‐report measure comprised of nine items that assess depressive symptoms (Kroenke et al. 2001). Items are rated on a 4‐point Likert scale ranging from 0 to 3, with scores summed to produce a total score. Higher totals indicate more severe depressive symptoms. The measure has demonstrated strong internal consistency (*α* = 0.88; Wilson, Krishnamorthy, et al. 2025).

#### 2.3.5 Child Anxiety Scale

The CAS‐8 is a self‐report questionnaire measuring generalised anxiety symptoms (Reardon et al. 2018). It is scored from 8‐items on a 4‐point Likert scale with higher scores indicating greater generalised anxiety symptoms and has demonstrated excellent internal reliability (*α* = 0.96; Wilson, Krishnamorthy, et al. 2025).

#### 2.3.6 Social Anxiety (SCAS)

The social anxiety subscale from the Spence Children's Anxiety Scale (Spence et al. 2003) was used to measure social anxiety symptoms. The scale is comprised of five items scored on a 4‐point scale, with higher scores reflective of higher social anxiety, and has demonstrated strong internal consistency (*α* = 0.81; Wilson, Krishnamorthy, et al. 2025).

#### 2.3.7 Obsessive Compulsive Inventory—Child Version

The OCI‐CV‐R is an 18‐item self‐report measure of obsessive‐compulsive disorder (OCD) symptoms (Abramovitch et al. 2022; Foa et al. 2010). Responses range from 0 (never) to 2 (always). Scores are summed to form a global score, which has demonstrated strong internal consistency (*α* = 0.93; Wilson, Krishnamorthy, et al. 2025).

#### 2.3.8 Borderline Personality Features Scale for Children ‐ 11 (BPFSC‐11)

The BPFSC‐11 is an 11‐item self‐report questionnaire measuring borderline personality symptoms (Sharp et al. 2014). Responses are rated on a 5‐point scale. Scores are summed to form a global score (11–55), and it has demonstrated strong internal consistency *α* = 0.92 (Wilson, Krishnamorthy, et al. 2025).

### 2.4 Ethics

The project was approved by the Children's Health Queensland research ethics committee (HREC/20/QCHQ/67708), with consent obtained from parents/legal guardians as per ethical approval.

### 2.5 Statistical Analysis

SPSS version 31 was used to analyse all data. Binary logistic regression was used to test the predictors effect on dropout, which was operationalised as not completing full course of FBT treatment for any reason. Linear regression was used to test the same predictor variables on EOT EDE‐Q global score. Predictors in all models included age, initial BMI centile, and baseline EDE‐Q, CAS, SAD, PHQ9, OCI‐CV, and BPFS scores. For this outcome, analyses were performed for the pooled cohort (i.e., combined AN and AAN), due to recent evidence showing minimal differences between AN and AAN in response to ED psychopathology across FBT (Urban et al. 2025), and that there were no baseline differences between any variables (except BMI) or drop‐out rates between the AN and AAN groups, and no difference to the interpretation of the results when controlling for diagnosis (NOTE: linear regression predicting BMI outcomes for the pooled group are shown in Supporting Information S1). Pre‐ and post‐treatment differences on BMI Centiles, ED and comorbid psychopathology are presented with repeated measures *t*‐tests reported.

For the AN group only, additional outcomes of “Full response” was calculated as achieving both ‘good’ EDE‐Q outcome (EDE‐Q global score within one standard deviation from community mean < 2.77 (Mond et al. 2006), and above the 25^th^ BMI centile at end of treatment (EOT), which are consistent with outcome measures used previously (Calugi, Cattaneo, et al. 2025; Calugi, Chimini, et al. 2025; Le Grange et al. 2019; Robin et al. 1999) (NOTE: Drop‐out and EDE‐Q outcomes are also shown for the AN cohort for completeness).

To address missing data for BMI centile and EDE‐Q global scores, multiple imputation using the fully conditional specification method with the Predictive Mean Matching procedure was used (Takahashi 2017), which is suitable for arbitrary missing data patterns. At EOT, the proportion of missing data was 1.5% for BMI centile, 50.3% for EDE‐Q.

Little's MCAR test showed that the pattern of missingness was missing completely at random (*χ* ^2^ = 0.246, df = 1, *p* = 0.62). Independent *t*‐tests comparing missing versus complete data for baseline variables showed no significant results, which is consistent with data missing at random, or completely at random assumptions, justifying the use of multiple imputation. Five imputed datasets were used with descriptive statistics stable across all. An a priori power analysis for logistic regression estimated that a total sample size of 130 would provide 80% power to detect an odds ratio of 2.0 with a significance level of *α* = 0.05, which was based off estimated group sample proportions from Bentz et al. (2025). Due to the exploratory nature of the study, no corrections for multiple comparisons were made, with predictors tested at the *p* < 0.05 level.

## 3 Results

### 3.1 AN and AAN Cohort

Means, standard errors, and mean difference confidence intervals are shown in Table 1. Results showed significant mean differences from pre to post FBT on all variables. Table 2 shows the bivariate logistic regression predicting drop out. Age was the only significant predictor, with older age predicting a greater likelihood of drop out from FBT. The percentage of each age group completing FBT are displayed in Table 3, illustrating the increased frequency of drop out as age increases. Similarly, as shown in Table 4, the linear regression analyses for EOT EDE‐Q revealed that baseline EDE‐Q was the only significant predictor, with higher baseline predicting higher EOT scores.

**TABLE 1:** Means, standard errors and confidence intervals for mean differences for pre and post intervention measures for AN and AAN.

Independent variable (range)	Pre (SE)	Post (SE)	*p*	95% CI	
				Lower	Upper
BMI centile	38.19 (2.44)	60.79 (2.05)	< 0.01	−26.50	−18.69
EDE‐Q (0–6)	3.75 (0.14)	1.54 (0.19)	< 0.01	1.78	2.62
Depression (0–27)	14.54 (0.85)	9.09 (0.98)	< 0.01	3.69	7.21
Generalised anxiety (0–24)	14.09 (0.73)	10.25 (0.84)	< 0.01	2.19	5.47
Social anxiety (0–18)	10.98 (0.58)	8.66 (0.65)	< 0.01	1.16	3.89
Obsessive compulsive symptoms (0–42)	14.84 (1.26)	11.89 (1.48)	< 0.01	0.79	2.67
Borderline personality features (11–55)	30.43 (1.23)	26.76 (1.68)	< 0.01	0.99	2.68

*Note:* Pooled data are presented.

Abbreviations: BMI, Body Mass Index; EDE‐Q, Eating Disorder Examination Questionnaire; SE, standard error.

**TABLE 2:** Logistic regression analysis of drop out in FBT with adolescents with AN and AAN.

Predictor variables	Dependent variable: Drop‐out			
	β	*p*	OR	95% CI OR
**Age**	**0.30**	**0.04**	**1.35**	**1.02–1.80**
BMI centile	−0.34	0.66	0.71	0.16–3.22
EDE‐Q	0.17	0.34	1.18	0.84–1.66
Depression	0.01	0.87	1.01	0.91–1.11
Generalised anxiety	0.02	0.81	1.02	0.90–1.15
Social anxiety	−0.06	0.41	0.94	0.82–1.09
Obsessive compulsive symptoms	−0.01	0.68	0.99	0.93–1.05
Borderline personality features	0.05	0.12	1.05	0.99–1.12

*Note:* Drop out coded: 1 = completed treatment, 2 = did not complete treatment. Pooled data are presented. EDE‐Q and BMI at baseline was used as predictor. Bold text denotes significant at *p* < 0.05.

Abbreviations: BMI, Body Mass Index; EDE‐Q, Eating Disorder Examination Questionnaire.

**TABLE 3:** Percentage of each age completing FBT.

Age (years)	Completed FBT	Started FBT	Percentage completed
11	4	7	57%
12	6	8	75%
13	14	27	52%
14	14	28	50%
15	14	31	45%
16	9	26	35%
17	3	8	38%

**TABLE 4:** Linear regression analysis of end of treatment EDE‐Q in FBT with adolescents with AN and AAN.

Predictor variables	Dependent variable: EDE‐Q score at EOT				
	β	*t*	*p*	95% CI for *β*	
				Lower	Upper
Age	−0.17	−1.51	0.13	−0.39	0.52
BMI centile	−0.72	−0.81	0.43	−2.66	1.22
**EDE‐Q**	**0.70**	**5.08**	**<** **0.01**	**0.43**	**0.98**
Depression	−0.07	−1.38	0.19	−0.18	0.04
Generalised anxiety	0.02	0.35	0.73	−0.10	0.13
Social anxiety	0.01	0.15	0.88	−0.11	0.13
Obsessive compulsive symptoms	−0.02	−0.64	0.53	−0.08	0.04
Borderline personality features	0.05	1.73	0.09	−0.01	0.11

*Note:* Pooled data are presented. EDE‐Q and BMI at baseline was used as predictor. Bold text denotes significant at *p* < 0.05.

Abbreviations: BMI, Body Mass Index; EDE‐Q, Eating Disorder Examination Questionnaire.

### 3.2 AN Group Only

Means, standard errors, and mean difference confidence intervals are shown in Table 5. Results showed significant mean differences from pre to post FBT on all variables. Tables 6 and 7 show the bivariate logistic regressions predicting drop out and full response outcome, with no significant predictors identified. For EOT BMI centile, age and baseline BMI were both significant predictors, with older age predicting lower EOT BMI centile, and higher baseline BMI centile predicting higher EOT BMI centile, as shown in Table 8. The linear regression analyses for EOT EDE‐Q found no significant predictors, as shown in Table 9.

**TABLE 5:** Means, standard errors and confidence intervals for mean differences for pre and post intervention measures for AN group only.

Predictor variable (range)	Pre (SE)	Post (SE)	*p*	95% CI	
				Lower	Upper
BMI centile	10.55 (1.91)	47.97 (3.79)	< 0.01	−43.83	−30.99
EDE‐Q (0–6)	3.49 (0.24)	1.41 (0.35)	< 0.01	1.29	2.87
Depression (0–27)	14.95 (1.59)	9.13 (1.7)	< 0.01	2.57	9.09
Generalised anxiety (0–24)	14.59 (1.59)	10.23 (1.48)	< 0.01	1.62	7.11
Social anxiety (0–18)	10.86 (1.06)	8.68 (1.14)	0.04	0.15	4.21
Obsessive compulsive symptoms (0–42)	14.90 (2.28)	9.10 (2.26)	< 0.01	2.72	8.90
Borderline personality features (11–55)	29.81 (2.03)	23.23 (2.74)	< 0.01	2.04	11.11

*Note:* Pooled data are presented.

Abbreviations: BMI, Body Mass Index; EDE‐Q, Eating Disorder Examination Questionnaire; SE, standard error.

**TABLE 6:** Logistic regression analysis of drop out in FBT with adolescents with AN only.

Predictor variables	Dependent variable: Drop‐out			
	β	*p*	OR	95% CI OR
Age	0.14	0.58	1.15	0.71–1.87
BMI centile	−1.34	0.67	0.26	0.01–129.47
EDE‐Q	−0.20	0.57	0.82	0.42–1.60
Depression	−0.08	0.34	0.92	0.78–1.09
Generalised anxiety	0.27	0.15	1.31	0.91–1.87
Social anxiety	−0.12	0.41	0.89	0.67–1.18
Obsessive compulsive symptoms	−0.02	0.74	0.98	0.87–1.10
Borderline personality features	0.10	0.16	1.11	0.96–1.27

*Note:* Drop out coded: 1 = completed treatment, 2 = did not complete treatment. Pooled data are presented. EDE‐Q and BMI at baseline was used as predictor.

Abbreviations: BMI, Body Mass Index; EDE‐Q, Eating Disorder Examination Questionnaire.

**TABLE 7:** Logistic regression analysis of full response in FBT with adolescents with AN only.

Predictor variables	Dependent variable: Full response			
	β	*p*	OR	95% CI OR
Age	0.05	0.86	1.05	0.61–1.82
BMI centile	−0.80	0.86	0.45	0.01–45.83
EDE‐Q	0.18	0.65	1.20	0.54–2.68
Depression	0.04	0.67	1.04	0.86–1.26
Generalised anxiety	−0.02	0.90	0.98	0.73–1.31
Social anxiety	0.05	0.69	1.05	0.82–1.35
Obsessive compulsive symptoms	0.05	0.49	1.05	0.91–1.21
Borderline personality features	−0.01	0.87	0.99	0.86–1.14

*Note:* Full response = < 2.77 EDE‐Q global score and BMI centile > 25^th^ at EOT, coded as: Full response = 1, Not full response = 2. Pooled data are presented. EDE‐Q and BMI at baseline was used as predictor.

Abbreviations: BMI, Body Mass Index; EDE‐Q, Eating Disorder Examination Questionnaire.

**TABLE 8:** Linear regression analysis of end of treatment BMI Centile in FBT with adolescents with AN only.

Predictor variables	Dependent variable: BMI centile at EOT				
	β	*t*	*p*	95% CI for *β*	
				Lower	Upper
**Age**	**−0.06**	**−2.53**	**0.01**	**−0.11**	**−0.01**
**BMI centile**	**0.86**	**3.04**	**<** **0.01**	**0.31**	**1.42**
EDE‐Q	0.02	0.58	0.56	−0.05	0.08
Depression	0.01	0.24	0.81	−0.01	0.02
Generalised anxiety	−0.01	−0.23	0.82	−0.03	0.02
Social anxiety	−0.01	−0.52	0.61	−0.03	0.02
Obsessive compulsive symptoms	−0.01	−0.44	0.66	−0.01	0.01
Borderline personality features	0.01	−0.03	0.98	−0.01	0.01

*Note:* Pooled data are presented. EDE‐Q and BMI at baseline was used as predictor.

Abbreviations: BMI, Body Mass Index; EDE‐Q, Eating Disorder Examination Questionnaire.

**TABLE 9:** Linear regression analysis of end of treatment EDE‐Q score in FBT with adolescents with AN only.

Predictor variables	Dependent variable: EDE‐Q global at EOT				
	β	*t*	*p*	95% CI for *β*	
				Lower	Upper
Age	0.03	0.13	0.90	−0.41	0.46
BMI centile	1.43	0.59	0.56	−3.51	6.36
EDE‐Q	0.43	1.69	0.09	−0.07	0.92
Depression	0.02	0.30	0.77	−0.19	0.16
Generalised anxiety	−0.05	−0.41	0.69	−0.28	0.19
Social anxiety	−0.01	−0.01	0.99	−0.23	0.22
Obsessive compulsive symptoms	0.07	1.40	0.17	−0.03	0.17
Borderline personality features	−0.05	−0.90	0.37	−0.15	0.05

*Note:* Pooled data are presented. EDE‐Q and BMI at baseline was used as predictor.

Abbreviations: BMI, Body Mass Index; EDE‐Q, Eating Disorder Examination Questionnaire.

## 4 Discussion

This study aimed to investigate predictors of FBT outcomes for adolescents with AN and AAN in a real‐world public community setting. Specifically, it aimed to examine whether patient‐level comorbid symptomology predicted drop‐out, responses to treatment and EOT BMI and EDE‐Q scores.

Results showed patient age significantly predicted drop out, with older adolescents less likely to complete FBT. This could be due to a number of reasons. It could be that family units with older adolescents (who may have age‐appropriate autonomy and be more individuated prior to ED onset) find it more challenging to have their parents in control of their eating during early FBT. On the other hand, although not measured in the current study, older adolescents may be more likely to have a longer duration of illness, which is associated with poorer treatment outcomes and could also explain this result (Treasure et al. 2015).

Alternatively, in the current setting, patients experiencing difficulties in FBT are routinely referred to CBT‐E and/or FBT‐informed Day Programme, with good effect (Wilson, Loxton, et al. 2025; Wilson, Withington, et al. 2025). This practice may lead to a clinic‐specific lower threshold for transitioning to other treatments rather than persisting with FBT, especially for older adolescents and families who may have a desire for more autonomy in their treatment, consistent with previous findings that older adolescents are more likely to choose CBT‐E over FBT Le Grange et al. (2020). The clinic‐specific effects are supported by the slightly higher rates of drop‐out than have been reported in similar settings (35% in Le Grange et al. 2020), although there is evidence of insufficient reporting/consideration of drop‐out in reviews of existing literature, which may bias existing estimates (Austin et al. 2025).

Non‐ED comorbid symptomology was not associated with EOT EDE‐Q scores, with baseline scores on the same measure emerging as the only significant predictor. For EOT BMI centile, age and baseline BMI centile were predictors with older age associated with lower EOT BMI centile, which is consistent with previous results (Le Grange et al. 2020) and supports the drop‐out findings from the current study. For the AN only cohort, the only significant predictors across the analyses were age and baseline BMI centile predicting EOT BMI centile.

The non‐significant findings with respect to non‐ED comorbid psychopathology are consistent with some of the previous literature (e.g., Datta et al. 2023), however contrasts with recent CBT‐E findings in the same setting, which found generalised anxiety symptoms was a predictor of drop out (Wilson et al., under review). This could reflect the differences in emphasis between the treatments (i.e., parent vs. young person), as it may be that parent‐level factors are more important in FBT, whereas patient‐level factors are more at play in CBT‐E. This is supported by the body of work in FBT that highlights the role of the family environment and expressed emotion impacting treatment outcome (Gorrell et al. 2022). The current findings implicate older age as a potential barrier to FBT's effectiveness, which warrants further consideration. It could be that modifications to FBT may be required to better tailor treatment to older age groups and overcome barriers to drop‐out and weight regain, in line with evidence in support of FBT for transition‐age youth (16–25 years; Dimitropoulos et al. 2018). Another potential implication could be for older adolescents to intensify FBT (e.g., through day programme treatment, multiple family meals at treatment onset, additional supports for meal preparation and supervision) from the start of treatment to achieve early gains as quickly as possible, minimising potential for future drop‐out. Additionally, in line with evidence supporting early intervention models (Allen et al. 2020), it may be warranted to consider prioritising younger cohorts for FBT in clinical settings, with individual‐focused treatment potentially better suited to older adolescents, which is tentatively supported by findings that older low‐weight adolescents that engaged with CBT‐E gain more weight than their younger counterparts (Le Grange et al. 2020). However, further, well‐controlled, research is required to confirm such hypotheses.

Finally, treatment effects for all outcome variables highlights the effectiveness of FBT in a real‐world setting among a cohort with significant comorbid symptomology. The findings that all non‐ED comorbid symptomology reduced after FBT treatment, potentially highlights the effects of targeting core ED symptoms resulting in improvements to secondary ED symptoms that may be due to starvation syndrome or psychosocial impairment due to the ED (e.g., Keyes 1950).

The strengths of the current study include its real word setting, large sample size (for this cohort), use of well validated comorbidity measures, and implementation of manualised FBT by well‐trained clinicians in a setting with high patient complexity. The lack of follow up data prevents understanding of the sustainability of treatment effects, or any predictor effects that may become evident at post‐treatment. Duration of illness of not controlled for, which may be a confounder for the results related to age, although this may be less of a factor in child and youth versus adult populations. The 25^th^ centile was used as indicator of full response in the AN in line with previous results (Calugi, Cattaneo, et al. 2025; Calugi, Chimini, et al. 2025; Le Grange et al. 2019; Robin et al. 1999), however there are a range of weight outcomes used in the literature to indicate remission, and the results of the current study may be different if different values were used (Le Grange et al. 2019). There was no comparison group, which prevents attribution of treatment or predictor effects to the intervention. The sample had limited diversity information collected (e.g., no race or ethnicity), and most participants were female, which limits generalisability to more diverse populations.

## 5 Conclusions

The current study affirmed the effectiveness of FBT for adolescents with AN and AAN, who showed improvement on BMI outcome, as well as ED and comorbid psychopathology. When accounting for a wide range of comorbid symptomology (depression, generalised anxiety, social anxiety, obsessive compulsive disorder, and borderline personality disorder symptoms), we found that age was a significant predictor of poor FBT outcomes, with older patients showing higher rates of drop‐out and lower EOT BMI. This highlights further considerations may be required for older adolescents to optimise outcome. Future research should replicate the current findings in a more controlled setting including follow up data to further establish these findings.

## Author Contributions

D.W. conceptualised the study, analysed the data and wrote the original draft. N.L. supervised methodology and conceptualisation and contributed to the writing, reviewing and editing of the manuscript. R.A.M. contributed the writing, reviewing and editing of the manuscript and data curation. J.W., R.L., J.I., J.A. were involved with conceptualisation and investigation, and contributed to reviewing and editing the manuscript. All authors read and approved the final manuscript.

## Funding

D.W. is supported by a Queensland Health Clinical Research Fellowship. The funder has no role in the conceptualization, design, data collection, analysis, decision to publish, or preparation of the manuscript.

## Ethics Statement

The project was approved by the Children's Health Queensland Hospital and Health Service Human Research Ethics Committee (EC00175) HREC/20/QCHQ/67708. Informed consent was obtained from parents/legal guardians as per ethical approval.

## Consent

The authors have nothing to report.

## Conflicts of Interest

The authors declare no conflicts of interest.

## Supporting information


Supporting Information S1

## Data Availability

The data that support the findings of this study are available on request from the corresponding author. The data are not publicly available due to privacy or ethical restrictions.
