# Supplementary material for: Examining Comorbid Psychopathology Symptoms as Predictors of Family Based Treatment for Adolescents With Anorexia Nervosa and Atypical Anorexia Nervosa in a Real‐World Setting
Source: Eur Eat Disord Rev. 2026 May 2;34(5):1360–9. doi: 10.1002/erv.70124 (PMC13432450; doi:10.1002/erv.70124)
Supplement: Supplementary file 1 — Supporting Information S1 [file ERV-34-1360-s001.docx]

Supplemental 1. Linear regression analysis of end of treatment BMI Centiles in FBT with adolescents with AN and AAN.

| Predictor variables | Dependent variable: BMI Centile at EOT | | | | |
| --- | --- | --- | --- | --- | --- |
|  | β | *t* | *p* | 95% CI for β | |
|  |  |  |  | Lower | Upper |
| **Age** | **-.03** | **-2.34** | **.02** | **-.05** | **-.01** |
| **Body mass index centile** | **.50** | **7.24** | **<.01** | **.37** | **.64** |
| EDE-Q | .02 | 1.42 | .16 | -.01 | .05 |
| Depression | -.01 | -1.06 | .29 | -.01 | .01 |
| Generalised Anxiety | .01 | .75 | .45 | -.01 | .02 |
| Social Anxiety | -.01 | -1.25 | .21 | -.02 | .01 |
| Obsessive Compulsive Symptoms | .01 | .42 | .68 | -.01 | .01 |
| Borderline Personality Features | -.01 | -.54 | .59 | -.01 | .01 |

Note: Pooled data are presented. EDE-Q = Eating Disorder Examination Questionnaire. EDE-Q baseline score was used as predictor.
